# Pan-immune-inflammation value: racial variations and differences in prognostic accuracy across breast cancer subtypes at a single institution

**DOI:** 10.3389/fonc.2026.1694711

**Published:** 2026-03-06

**Authors:** Indigo Johnson, Atif Bacchus, Ross Budziszewski, Scott D. Siegel, Jennifer Sims-Mourtada

**Affiliations:** 1 Cawley Center for Translational Cancer Research, Helen F. Graham Cancer Center & Research Institute, ChristianaCare, Newark, DE, United States; 2 Philadelphia College of Osteopathic Medicine, Philadelphia, PA, United States; 3 Biological Sciences, The University of Delaware, Newark, DE, United States

**Keywords:** breast cancer, pan-immune-inflammatory value, prognostic biomarker, racial disparities, systemic inflammation, triple-negative breast cancer

## Abstract

**Introduction:**

The Pan-Immune-Inflammatory-Value (PIV) has shown promise as a biomarker for predicting survival outcomes in breast cancer (BC) patients. This study explores the variation of PIV across BC subtypes, with a focus on triple-negative BC (TNBC), hormone receptor positive and negative (HR+ and HR-) cancers, and racial disparities in immune response.

**Methods:**

A retrospective review of laboratory and clinical data of 2,597 BC patients treated between 2012–2022 was conducted. PIV was calculated as a composite marker of neutrophils, monocytes, lymphocytes, and platelets. Comparative analysis of PIV with race, subtype and stage was performed using Man-Whitney. For categorical analysis of PIV, receiver operating characteristic curve was generated, and the optimal cutoff point was determined using the Youden index (cutoff = 395). Descriptive and inferential tests were used to compare high/low PIV groups based on race and BC subtype. Disease-free survival (DFS), and overall survival (OS) were evaluated with Kaplan-Meier curves and Cox regression analysis.

**Results:**

PIV varied significantly by race and subtype; Black women were significantly less likely to present with high PIV (OR = 0.595, 95% CI: 0.505 – 0.701) compared to White women. Lower values were also observed in Black women, TNBC patients and those with HR− tumors. Over a median follow-up of 55.4 months, high PIV was associated with worse DFS and OS in the overall cohort (p < 0.0005). Subgroup analysis showed high PIV predicted shorter DFS in both Black and White women but was not associated with OS in Black women or DFS in TNBC. In multivariable Cox regression, stage and PIV independently predicted DFS and OS. Significant interactions were observed between PIV and both race and subtype for breast cancer outcomes.

**Conclusion:**

While PIV shows promise as a general prognostic indicator, its predictive accuracy varies across different receptor subtypes. In HR+ BC patients, PIV is linked to clinical outcomes, supporting its role as a prognostic biomarker. However, further research is needed to assess the use of PIV as a prognostic biomarker.

## Introduction

Breast cancer (BC) is the most common non-dermatologic malignancy in women and accounts for approximately 42,500 deaths annually in the United States (1). Despite similar incidence rates between Black and White women, Black women have a 41% higher mortality rate compared to White women (2). This disparity is particularly evident in triple-negative BC (TNBC), a highly aggressive subtype (3, 4), which is typically characterized by high-grade tumors that grow and spread rapidly, leading to increased mortality rates. TNBC disproportionately impacts Black women, who are twice as likely to develop this subtype (5, 6). The combination of its elevated prevalence, limited treatment options, and poor prognosis emphasizes the need for more effective therapeutic strategies and personalized approaches to care. While advancements in screening and treatments have reduced overall BC mortality (7, 8), significant variations in treatment response and prognosis persist, even among patients with similar cancer types and stages. This variability highlights the critical need for further research into prognostic indicators that can more accurately predict treatment outcomes and guide clinical decision-making.

Inflammation, while essential for the body’s natural defense against injury and infection, can also create a microenvironment that facilitates tumor growth and metastasis. Inflammation is a well-known hallmark of cancer, playing a critical role in tumor promotion and progression through processes such as angiogenesis, inflammatory cell recruitment, tissue remodeling, and invasion (9–12). The propagation of pro-inflammatory cytokines, growth factors, and chemokines promote a state of equilibrium between inflammation and tumor progression (13, 14). Immune cells recruited to the site of inflammation can undergo functional reprogramming, shifting from tumor-suppressive to tumor-promoting roles (13). Inflammation and cancer progression call for a broader assessment of the immune-inflammatory landscape, beyond what traditional biomarkers have previously captured.

Previous biomarkers such as the monocyte-to-lymphocyte ratio (MLR), platelet-to-lymphocyte ratio (PLR), and neutrophil-to-lymphocyte ratio (NLR) which are derived from routine complete blood counts with differentials test, have shown promise in predicting neoadjuvant chemotherapy responses across various solid tumors (15–17). However, there is a growing need for more robust and comprehensive modalities that can evaluate the immune landscape and predict chemotherapy and radiotherapy response more effectively. One such metric is the Pan-Immune-Inflammatory Value (PIV), a composite biomarker that integrates neutrophil, platelet, monocyte, and lymphocyte counts to provide a more comprehensive measure of systemic inflammation and immune status. While NLR, PLR, and MLR provide prognostic value, they each focus on limited immune components, failing to capture the full scope of systemic inflammation. PIV overcomes these limitations by considering platelet count as well, offering a broader and more integrated assessment of immune-inflammatory activity. By providing a more holistic evaluation than traditional markers, PIV enhances the accuracy of survival predictions (17, 18). A recent meta‐analysis of eight breast cancer studies involving 2,953 patients found that high PIV was associated with a significantly increased risk of death (HR = 2.045, 95% CI: 1.355–3.086, p = 0.001) (17, 18). These consistent results across multiple studies suggest that PIV could be utilized as a valuable prognostic tool. Moreover, similar associations have been reported in other cancer types such as colorectal cancer (19–21), suggesting that PIV may serve as a tool across a broad spectrum of malignancies.

While PIV shows promise as a prognostic biomarker, it remains unclear whether its utility applies across BC subtypes. The objective of this study was to investigate the role of race and receptor status in influencing the predictive utility of PIV in patients diagnosed with BC. Given the well-documented racial disparities in BC outcomes, with Black patients experiencing a higher prevalence of TNBC (2, 22, 23), and displaying differences in tumor infiltrating immune responses than White patients (24–26), we sought to evaluate the effectiveness of PIV as a prognostic tool across Black and White racial groups. We explored the interaction between receptor status and immune-inflammatory composition to assess PIV’s prognostic significance across hormone receptor (HR) and TNBC subtypes.

## Materials and methods

### Study design and population

This single-site, retrospective study reviewed clinical and laboratory data from 2,597 patients diagnosed with BC or receiving treatment at the ChristianaCare Helen F. Graham Cancer Center & Research Institute (HFGCCRI) between January 2012 and December 2022. This study was approved by the ChristianaCare Institutional Review Board (CCC#35082). Patients were identified through the HFGCCRI Cancer Registry and laboratory data were manually abstracted using patient electronic health records (EHR). Eligibility criteria for inclusion were: (a) female, (b) confirmed invasive breast adenocarcinoma diagnosis via pathological assessment, (c) treatment received at ChristianaCare, and (d) availability of data within six months, preferentially preceding patient’s diagnosis. Patients who did not meet these criteria were excluded (**Supplementary Figure 1**). Due to disruptions in clinical workflows, and recordkeeping associated with COVID-19 restrictions, patients who were diagnosed between 2020–2021 were excluded from this study.

### Data collection and definitions

Age, race, tumor staging, histological subtypes, data on survival duration, and vital status were obtained from the ChristianaCare cancer registry. For the focus of our study, race was defined as White and Black. All the other races were excluded to avoid inaccurate results due to small sample size. CBC with differentials data within six months of diagnosis were abstracted from ChristianaCare’s EHR. This information was entered and managed using ChristianaCare’s Research Electronic Data Capture (REDCap) software (27). For each patient, the PIV immune-inflammatory marker was calculated from the CBC data using absolute values:


$$PIV=\;\frac{\text{Neutrophil count }(10^{9}/\text{L})\;\times \text{ Monocyte count }(10^{9}/\text{L})\;\times \text{ Platelet count }(10^{9}/\text{L})}{\text{Lymphocyte count }\times (10^{9}/\text{L})}$$

### Statistical analysis

Descriptive statistics were applied to summarize continuous variables, which are reported as means and standard deviation, and categorical variables, reported as frequencies and percentages. Comparative analysis of PIV with race, and subtype was performed using Mann-Whitney. For categorical analysis of PIV, the optimal PIV cut-off value was determined with receiver operating characteristic (ROC) curve analysis based on disease-free survival. The cut-off value of 395 was identified using Youden’s index (**Supplementary Figure 2**). This cut off is similar to that found in previous studies (28, 29). This threshold was utilized to categorize patients into high- and low-PIV groups (PIV score). Chi-square tests were used to evaluate associations between PIV groups and clinicopathological features and race. Mean differences were assessed using a two-tailed Student’s t-test. Survival analyses were conducted using the Kaplan-Meier method to estimate overall survival (OS) and disease-free survival (DFS). Differences between survival curves were assessed using the log-rank test. Multivariable Cox proportional hazards regression was used to evaluate the association between PIV and DFS or OS. PIV was treated as a continuous variable and was mean-centered prior to inclusion in regression models to facilitate interpretation of interaction terms and to reduce multicollinearity between main and interaction effects. A stepwise modeling strategy was employed to evaluate whether the prognostic effect of PIV differed by patient race and tumor characteristics. In the initial model, established clinical covariates—including disease stage (localized vs advanced), race (Black vs White), breast cancer subtype (TNBC vs non-TNBC), and hormone receptor status (HR-positive vs HR-negative)—were entered simultaneously. In the second model, centered PIV was added as a main effect. In the final model, multiplicative interaction terms between centered PIV and race, HR status, and subtype (PIV × race, PIV × HR status, and PIV × subtype) were included to formally assess effect modification. Statistical significance was set at a two-tailed p-value of < 0.05. All statistical analyses were performed using IBM SPSS Statistics version 25.0 and survival curves were generated using Prism GraphPad version 10.0.

## Results

### Study population characteristics

**Supplementary Table 1** presents the demographic and clinical characteristics of the study population which included 2,597 patients. Of these, 23.5% (n = 611) were identified as Black, and 76.5% (n = 1986) identified as White with a mean age 61.75 years. The clinical staging distribution showed that 58.5% of patients were classified as non-advanced (Stage I), while 41.5% were advanced (Stage IIA and above) (31). Among the patients, 18% were classified as having TNBC and 81.3% as non-TNBC. In terms of HR status, 77.2% were HR+ and 22.8% were HR-. The median follow up time was 55.40 Months (range 1.02–139 months).

### Distribution of PIV across race, subtype and stage

**Figure 1:**
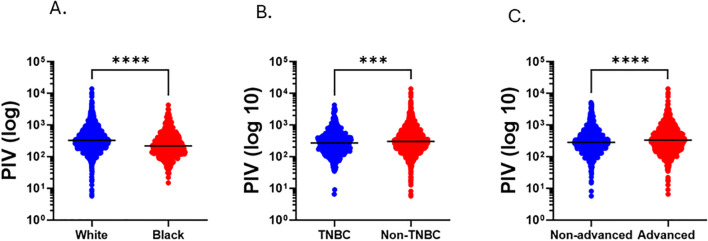
Distribution of PIV by race, subtype and stage. Violin plots comparing PIV scores across **(A)** race, **(B)** subtype and **(C)** stage. ***p < 0.005, ****p < 0.0005). Black line represents median score.

PIV differed significantly by race (**Figure 1**, **Table 1**). Black patients had lower PIV than White patients (p < 0.0005). This difference appears to stem from a higher absolute lymphocyte count in Black patients (p < 0.0005) alongside a lower absolute neutrophil (p < 0.0005) and monocyte (p < 0.0005) count (**Supplementary Table 2**).

**Table 1:** Comparison of Pan-Immune-Inflammatory-Value (PIV) groups (low versus high) stratified by race, cancer stage, receptor status, and hormonal receptor (HR).

Characteristics	Category	High PIV (n)	Low PIV (n)	Chi-Square (df)	OR (95% CI)^α^	p-value
Race	Black Patients	155	789	41.644 (1)	0.595 (0.505-0.701)	< 0.0005
	White Patients	1197	611			
Stage	Advanced	497	447	20.85 (1)	1.24 (1.133-1.359)	< 0.0005
	Non-Advanced	631	1022			
Subtype	TNBC	151	334	7.012 (1)	0.792 (0.665-0.943)	0.009
	Non-TNBC	334	2112			
Receptors	HR Positive	751	1254	4.656 (1)	0.847 (0.727-0.986)	0.032
	HR Negative	193	399			

^α^Reference categories are White Patients, Non-Advanced Stage, Non-TNBC, and HR Positive.

PIV was also significantly lower in patients with TNBC (p<0.005) compared with those who had non-TNBC tumors (**Figure 1**; **Table 1**; **Supplementary Table 2**). While lymphocyte counts did not differ between TNBC and non-TNBC groups (p = 0.466), TNBC cases had significantly lower absolute neutrophil (p < 0.0005) and monocyte counts (p < 0.0005). Similarly, patients with HR- tumors had lower PIV compared to those with HR+ (p < 0.0005), with significantly lower levels of absolute neutrophils (p < 0.0005) and absolute monocytes (p < 0.0005) with no significant differences observed in lymphocyte values (p = 0.622).

Patients with non-advanced stage BC had significantly lower PIV than those with advanced stage BC (**Figure 1**; **Table 1**; p < 0.0005). Patients with advanced stage BC had significantly higher levels of absolute neutrophils (p < 0.0005) and monocytes (p = 0.013) than non-advanced stage BC with no significant differences observed in lymphocyte levels (p = 0.081).

These findings underscore potential immune profile differences across both racial groups, tumor subtypes and stage, particularly due to variations in innate immune cell populations.

### Categorical analysis of high and low PIV groups by race, subtype and stage

High PIV has previously been associated with poor outcomes in BC (29, 30). A cut-off value for high PIV in our cohort was determined to be 395 for our study population (**Supplementary Figure 2**). In this cohort, 36.3% of patients were classified as high score (PIV > 395), and 63.7% were classified as low score (PIV ≤ 395). Across all patients, advanced stage cancers were strongly associated with high PIV score (**Table 2**; Chi-square = 20.850, p < 0.0005). Likewise, we found that White patients were significantly more likely to have high PIV scores (**Table 2**; Chi-square = 41.64, p < 0.0005). There was also a significant difference observed in PIV score based on BC subtypes (**Table 2**; Chi-square = 7.012, p = 0.009 for Non-TNBC vs. TNBC; Chi-square = 4.656, p = 0.017 for HR- vs. HR+).

**Table 2:** Stratified analysis comparing Pan-Immune-Inflammatory-Value (PIV) groups across cancer characteristics, stratified by race.

Characteristic	Race	High PIV (n)	Low PIV (n)	Chi-Square (df)	OR (95% CI)^α^	p-value
Stage: Non-Advanced	Black	80	244	12.056 (1)	0.647 (0.536-0.782)	< 0.001
	White	417	778			
Stage: Advanced	Black	64	279	27.887 (1)	0.499 (0.395-0.631)	< 0.005
	White	303	946			
Subtype: TNBC	Black	169	37	28.982 (1)	0.484 (0.359-0.653)	< 0.0005
	White	114	165			
Subtype: Non-TNBC	Black	287	118	15.119 (1)	0.684 (0.562-0.831)	< 0.0005
	White	1032	675			
Receptors: HR Positive	Black	107	272	24.636 (1)	0.657 (0.535-0.806)	< 0.0005
	White	982	644			
Receptors: HR Negative	Black	48	184	16.974 (1)	0.539 (0.413-0.704)	< 0.0005
	White	145	214			

^α^Reference category for all comparisons is White Patients.

### Racial differences in high and low PIV groups by stage

White patients were more likely to have high PIV and Black patients were more likely to have low PIV regardless of disease state (**Table 3**; Non-advanced Chi-Square 12.06, p < 0.001; Advanced Chi-Square = 27.89, p < 0.0005). Among White patients, those with advanced stage cancer were significantly more likely to have high PIV compared to those with non-advanced stage cancer (**Table 4**; Chi-square = 29.25, p < 0.0005). However, no significant differences in PIV scores were observed among Black patients when stratified by stage (**Table 3**; Chi-Square = 0.167, p = 0.71).

**Table 3:** Stratified analysis comparing Pan-Immune-Inflammatory-Value (PIV) groups across race, stratified by cancer stage, receptor status, and hormonal receptors (HR).

Race	Characteristic	High PIV (n)	Low PIV (n)	Chi-Square (df)	OR (95% CI)^α^	p-value
Black Patients	Stage					
	Advanced	75	244	0.167 (1)	1.041 (0.861-1.259)	0.71
	Non-Advanced	75	212			
	Subtype					
	TNBC	37	118	9.006 (1)	0.644 (0.475-0.874)	0.003
	Non-TNBC	169	287			
	Receptors					
	HR Positive	107	272	4.324 (1)	0.716 (0.531-0.966)	0.044
	HR Negative	48	184			
White Patients	Stage					
	Advanced	417	778	29.624 (1)	1.347 (1.210-1.499)	< 0.0005
	Non-Advanced	372	419			
	Subtype					
	TNBC	114	675	0.174 (1)	1.048 (0.895-1.308)	0.692
	Non-TNBC	165	1032			
	Receptors					
	HR Positive	982	644	0.055 (1)	1.023 (0.846-1.238)	0.812
	HR Negative	145	215			

^α^Reference categories are Non-Advanced (Stage), Non-TNBC (Subtype), and HR Positive (Receptor Status).

**Table 4:** Cox Regression modeling of disease-free survival.

Variable	Model 1			Model 2			Model 3		
	Exp(B)	95% CI	p-value	Exp(B)	95% CI	p-value	Exp(B)	95% CI	p-value
AJCC Stage	0.104	0.073-0.147	**0.000**	0.108	0.076 -0.152	**0.000**	0.107	0.0756-0.152	**0.000**
Age	1.011	1.003-1.020	0.100	1.010	1.001-1.019	0.250	1.010	1.001-1.019	0.222
Race	1.038	0.782-1.300	0.795	0.996	0.749-1.326	0.979	0.995	0.746-1.327	0.974
Subtype	0.769	0.427-1.384	0.381	0.761	0.422-1.371	0.363	0.537	0.267-1.080	0.081
HR Status	1.350	0.776-2.350	0.288	1.332	0.765-2.232	0.311	1.874	0.957-3.668	0.067
PIV	Not included	–	–	1.000	1.000-1.009	**0.000**	1.000	0.999-1.000	0.059
PIV*Race	Not included	–	–	Not included	–	–	1.001	1.000-1.001	**0.006**
PIV*Subtype	Not included	–	–	Not included	–	–	0.999	0.998-1.000	**0.005**
PIV*HR Status	Not included	–	–	Not included	–	–	1.001	1.000-1.002	**0.001**

Bold values indicate statistical significance (p < 0.05).

### Racial differences in PIV by breast cancer subtype

Significant racial differences in PIV classification were observed across BC subtypes. White patients had significantly higher PIV scores compared to Black patients across all BC subtypes. (**Table 3**; TNBC Chi-square = 28.982, p < 0.0005; Non-TNBC Chi-square = 15.119, p < 0.0005; HR- Chi Square = 16.974, p < 0.0005; HR+ Chi Square = 24.63, p <0.0005).

When comparing PIV classifications within each racial group, no significant differences were observed among White patients across BC subtypes. Specifically, PIV scores did not vary significantly between TNBC and Non-TNBC tumors or between HR+ and HR− tumors (**Table 3**; Chi-square = 0.174, p = 0.692; Chi-square = 0.055, p = 0.812). In contrast, significant differences were seen among Black patients, where those with TNBC or HR− tumors were more likely to be classified as low PIV compared to Black patients with non-TNBC tumors (**Table 3**; Chi-square = 9.006, p = 0.003). A smaller but still significant difference was also observed between HR+ and HR− tumors among Black patients, with lower PIV more common in HR− disease (**Table 3**; Chi-square = 4.324, p = 0.044).

### Association between PIV score and breast cancer outcomes

**Figure 2:**
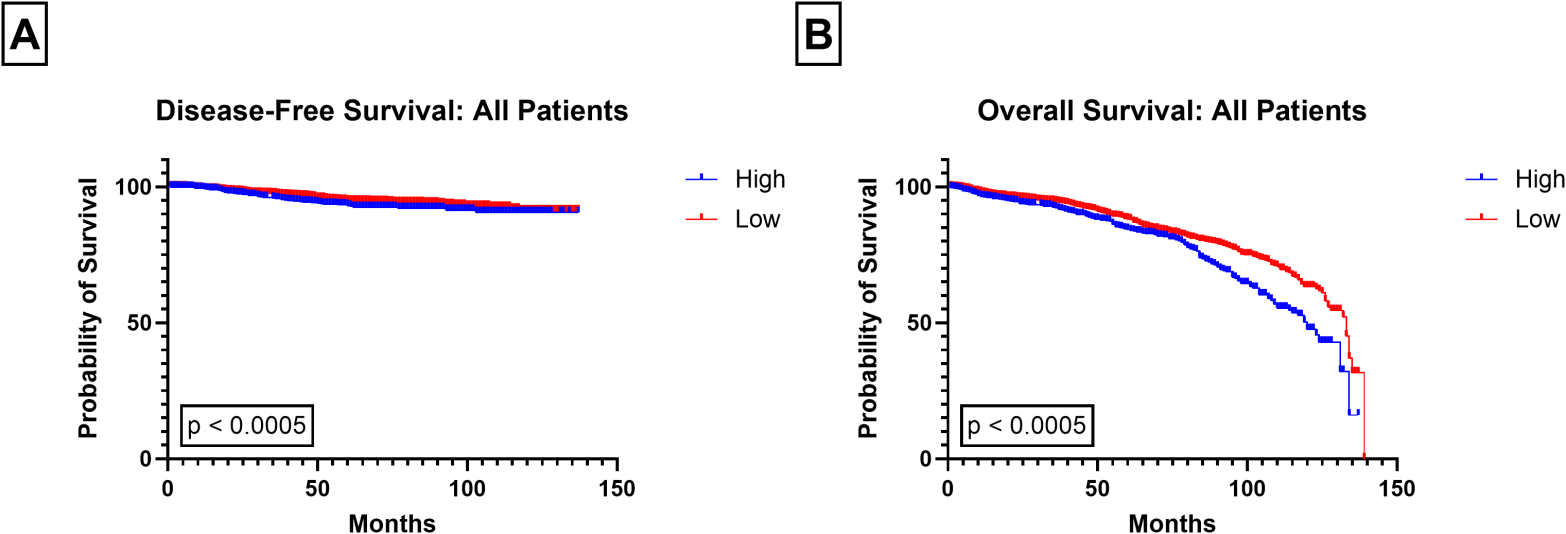
Kaplan-Meier plots of all patients. **(A)** Disease-Free Survival **(B)** Overall Survival in different Pan-Immune-Inflammatory-Value (PIV) groups (low versus high).

**Figure 3:**
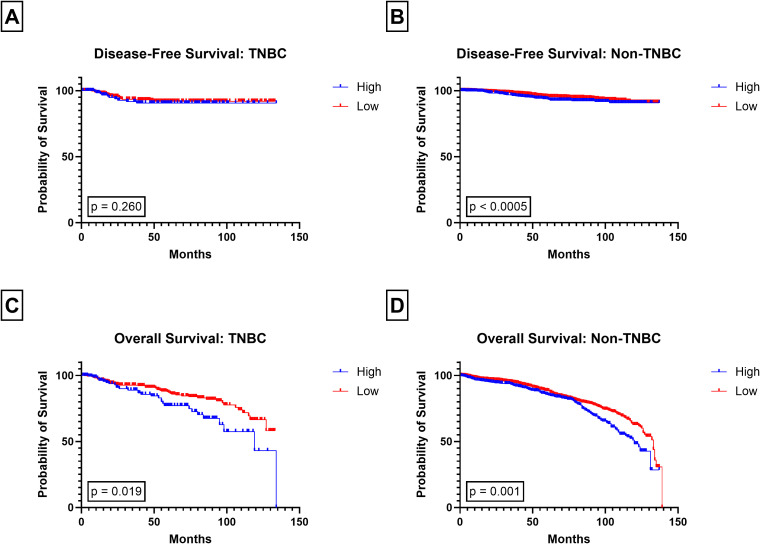
Kaplan-Meier plots of White vs Black patients. Disease-Free Survival in **(A)** White patients and **(B)** Black patients and Overall Survival in **(C)** White patients, and **(D)** Black patients, for low and high PIV groups.

High PIV scores were significantly associated with both DFS (p = 0.0005; **Figure 2A**) and OS (p < 0.0005; **Figure 2B**). Patients in the high PIV cohort had significantly shorter DFS times compared to those with low PIV, regardless of race (White p = 0.001; Black p = 0.001; **Figures 3A, B**). White patients with High PIV also had worse OS than those with low PIV (p < 0.0005; **Figure 3C**). However, there was no significant association between PIV levels and OS in Black patients (p = 0.988; **Figure 3D**). Similar findings were found when using a cut off value based on the median PIV value (**Supplementary Figures 3**, **4**).

**Figure 4:**
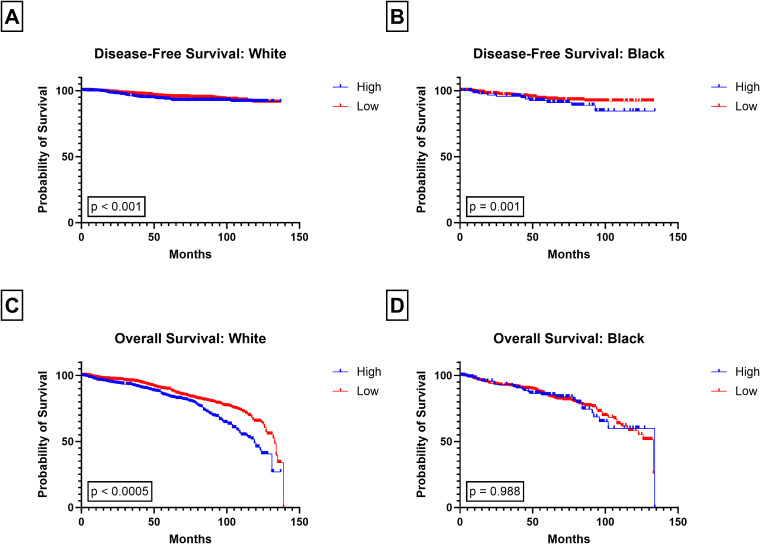
Kaplan-Meier plots of PIV stratified by tumor subtype. Disease-Free Survival in **(A)** TNBC and **(B)** Non-TNBC and Overall Survival in **(C)** TNBC, and **(D)** Non-TNBC, for low and high PIV groups.

**Figure 5:**
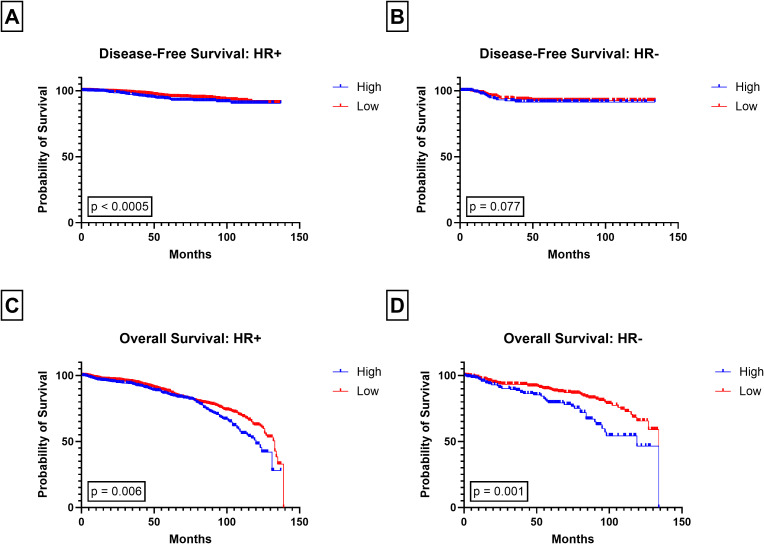
Kaplan-Meier plots of PIV stratified by hormone receptor status. Disease-Free Survival in **(A)** HR+ and **(B)** HR- and Overall Survival in **(C)** HR+, and **(D)** HR-, for low and high PIV groups.

While high PIV was significantly associated with worse DFS in non-TNBC patients (p < 0.0005; **Figure 4B**), no association was observed between high PIV and DFS in patients with TNBC (p = 0.260; **Figure 4A**). Similarly, High PIV was associated to poor survival in patients with HR+ tumors (p < 0.0005; **Figure 5A**) but not those with HR- tumors (p = 0.077, **Figure 5B**). High PIV was predictive of poor OS in all subtypes (**Figures 4**, **5**), although a stronger association was observed in non-TNBC than TNBC (p = 0.001 vs p = 0.019). High PIV was no longer significant when using the median PIV value as a cut off (**Supplementary Figure 5**).

To further explore whether the prognostic association of PIV varied across patient and tumor characteristics, multivariable Cox proportional hazards models were constructed for DFS and OS (**Table 4**; **Supplementary Table 3**). Models were constructed using a blockwise approach to evaluate the independent and context-dependent prognostic value of PIV. In the first block, established clinical and demographic covariates (age at diagnosis, stage, race, subtypes and HR status) were entered to account for baseline prognostic effects. In the second block, PIV was entered as a continuous variable. The final block included interaction terms (PIV × race, PIV × subtype, and PIV × HR status) to evaluate effect modification. Advanced stage was the strongest independent predictor of recurrence (p < 0.0005, **Table 4**). Age, race, breast cancer subtype, and hormone receptor status were not independently associated with DFS. When modeled as a continuous variable, higher PIV was significantly associated with worse DFS (p < 0.0005). However, inclusion of interaction terms revealed significant effect modification, with the association between PIV and DFS differing by race (PIV × race, p = 0.006), and breast cancer subtype (PIV × subtype, p = 0.005) and hormone receptor status (PIV × HR status, p = 0.001). Similar findings were observed with overall survival, where significant interaction was observed between PIV and subtype and HR status but not race (**Supplementary Table 3**.).

## Discussion

This study confirms previous findings that elevated PIV is associated with poorer breast cancer outcomes (28, 29). We further explored whether PIV’s prognostic value varies by race and BC subtype. Our findings show that Black women and patients with TNBC were more likely to exhibit lower PIV values and the prognostic impact of PIV was attenuated in these groups. Our data indicate that the prognostic signal of PIV is not uniform, supporting the need for caution when applying systemic inflammatory biomarkers across heterogeneous patient populations.

HR+ tumors are generally characterized by limited tumor-infiltrating lymphocytes and lower immune activation, whereas TNBC exhibit greater immune heterogeneity and, in some cases, higher levels of lymphocytic infiltration, which predict improved outcomes (30–34). In our cohort, TNBC patients exhibited lower PIV values driven by reduced neutrophil and monocyte counts and relatively higher lymphocyte levels. Yet this profile did not correspond to improved disease-free survival. Further research is needed to clarify how circulating immune profiles relate to the tumor immune microenvironment.

Black women in our cohort were significantly more likely to exhibit low PIV scores compared to White women, a pattern consistent across tumor subtypes and stages. This difference was primarily driven by lower absolute neutrophil and monocyte counts and higher lymphocyte levels among Black patients. When stratified by receptor status, we found that White patients with TNBC and HR− breast cancer were significantly more likely to have high PIV compared to Black patients. Interestingly, in HR+ breast cancer, no significant differences in PIV levels were observed between White and Black patients, suggesting that the influence of hormone receptor expression on systemic inflammation may be more uniform across racial groups. Additionally, prior studies in TNBC show that tumors from Black women exhibit higher lymphocyte infiltration, suggesting differences between tumor immune and circulating inflammatory profiles (24, 35–37). Genetic factors, including the Duffy-null polymorphism, prevalent among individuals of West African ancestry and associated with both benign ethnic neutropenia as well as TNBC, may contribute to low PIV values independent of tumor immune context (38, 39).

In our study, the relationship between PIV and outcomes depended heavily on how PIV was stratified, consistent with prior work showing prognostic separation by subtype only at the top quartile (40). Associations varied by race and subtype in both Kaplan–Meier and multivariable regression models. High PIV was linked to worse outcomes only in HR+ tumors, while results for race were mixed. Categorical PIV suggested racial differences in both DFS and OS, but continuous modeling showed no interaction between race and PIV in OS. Associations in TNBC also varied by PIV stratification. Importantly, Black women and those with TNBC were overrepresented in the low-PIV group, likely reflecting baseline differences and sensitivity to cut-off selection. Our findings suggest that continuous modeling approaches may be more accurate that stratification in heterogenous populations.

This study has several limitations. PIV was assessed retrospectively at a single time point near diagnosis using complete blood count data obtained without standardized timing. Because blood cell counts fluctuate over time, including with circadian rhythms, this approach may introduce measurement variability and limits assessment of immune dynamics. Prospective studies with standardized blood collection and longitudinal sampling will be needed to confirm these findings. As a single-institution analysis, generalizability may also be influenced by regional differences in patient demographics, healthcare access, and treatment practices. Although models were adjusted for clinicopathological factors, comorbidities and socioeconomic variables, which may differ by race and influence peripheral immune cell counts, were not available for adjustment.

In addition, a substantial proportion of patients were excluded due to missing or inconsistent clinical or laboratory data, as well as diagnoses during the COVID-19 pandemic, when disruptions in care delivery and laboratory availability were common. While these exclusions were necessary to ensure data integrity (**Supplementary Figure 1**), they may introduce selection bias by preferentially omitting patients with more complex clinical courses or differential inflammatory profiles. Moreover, despite excluding peak COVID-19 cases, pandemic-related factors such as subclinical infection or vaccination may still have influenced inflammatory markers. Finally, the study period spanned a decade during which breast cancer treatment paradigms evolved substantially, including the introduction of targeted therapies and immunotherapy, which may have influenced survival outcomes independently of baseline immune–inflammatory status. Together, these limitations indicate that subgroup findings should be interpreted as exploratory and warrant validation in larger, multi-institutional prospective studies.

Our findings highlight the potential utility and context-specific nature of PIV in breast cancer. While PIV showed prognostic associations in the overall cohort and in hormone receptor–positive disease, its performance varied by tumor subtype and race, with less consistent associations in triple-negative breast cancer and among Black women. These results suggest that the prognostic relevance of PIV may not be uniformly applicable across heterogeneous breast cancer populations.

## Data Availability

The datasets presented in this article are not readily available because Data sets will be made available under a data use agreement with the corresponding author. Requests to access the datasets should be directed to jsimsmourtada@christianacare.org.

## Supplementary material

Supplementary Table 1 Patient clinical characteristics.

Supplementary Table 2 Pan-Immune-Inflammatory-Value (PIV) and immune cell levels by patient characteristics. Immune cell values given at 10^3^ cells/µL.

Supplementary Table 3 Cox regression modeling of overall survival.

Supplementary Figure 1 Patient exclusion flowchart for cohort selection.

Supplementary Figure 2 Receiver operating characteristic curve (ROC) for optimal PIV cut-off threshold.

Supplementary Figure 3 Kaplan-Meier plots of in all patients with cut off based on median score. **(A)** Disease-Free Survival **(B)** Overall Survival in different Pan-Immune-Inflammatory-Value (PIV) groups (low versus high).

Supplementary Figure 4 Kaplan-Meier plots of in all patients by race with cut off based on median score. Disease-Free Survival, **(A)** White and **(B)** Black, and Overall Survival, **(C)** White and **(D)** Black in different Pan-Immune-Inflammatory-Value (PIV) groups (low versus high).

Supplementary Figure 5 Kaplan-Meier plots of in all patients by subtype with cut off based on median score. Disease-Free Survival, **(A)** TNBC and **(B)** Non-TNBC, and Overall Survival, **(C)** TNBC and **(D)** Non-TNBC in different Pan-Immune-Inflammatory-Value (PIV) groups (low versus high).
