# Supplementary material for: Pan-immune-inflammation value: racial variations and differences in prognostic accuracy across breast cancer subtypes at a single institution
Source: Front Oncol. 2026 Mar 6;16:1694711. doi: 10.3389/fonc.2026.1694711 (PMC13002395; doi:10.3389/fonc.2026.1694711)
Supplement: Supplementary file 1 [file Table1.docx]

| **Supplementary Table 1.**  **Patient clinical characteristics.** | |
| --- | --- |
| **Characteristics** | **Count** |
| **Age,** **Mean (SD)** | 61.75 (13.731) |
| **Race, *n* (%)** |  |
| Black Patients | 611 (23.5%) |
| White Patients | 1986 (76.5%) |
| **Stage, *n* (%)** |  |
| Advanced | 1078 (41.5%) |
| Non-Advanced | 1519(58.5%) |
| **Subtype, *n* (%)** |  |
| TNBC | 485 (18.7%) |
| Non-TNBC | 2112 (81%) |
| **Hormonal Receptors, *n* (%)** |  |
| Positive | 2005 (77.2%) |
| Negative | 592 (22.8%) |
| **Vital Status, *n* (%)** |  |
| Alive | 2127 (81.9%) |
| Deceased | 470 (18.1%) |
| **PIV Groups, *n* (%)** |  |
| High | 944 (36.3%) |
| Low | 1653 (63.7%) |
| **Total, *N* (%)** | 2597 (100) |
