# Supplementary material for: Pan-immune-inflammation value: racial variations and differences in prognostic accuracy across breast cancer subtypes at a single institution
Source: Front Oncol. 2026 Mar 6;16:1694711. doi: 10.3389/fonc.2026.1694711 (PMC13002395; doi:10.3389/fonc.2026.1694711)
Supplement: Supplementary file 2 [file Table2.docx]

**Supplementary Table 2.** Pan-Immune-Inflammatory-Value (PIV) and immune cell levels by patient characteristics. Immune cell values given at 10^3^ cells/µL.

|  | **PIV** | | | **Neutrophils** | | | **Monocytes** | | | **Lymphocytes** | | |
| --- | --- | --- | --- | --- | --- | --- | --- | --- | --- | --- | --- | --- |
|  | Median | Range | p-value | Median | Range | p-value | Median | Range | p-value | Median | Range | p-value |
| **Race** |  | | |  | | |  | | |  | | |
| Black | 219.69 | 12.00-4318.00 | < 0.0005 | 3.55 | 0.52-15.10 | < 0.0005 | 0.48 | 0.61-1.94 | < 0.0005 | 2.00 | 0.195-5.425 | < 0.0005 |
| White | 328.6 | 5.74-14002.02 |  | 4.42 | 0.64-16.00 |  | 0.56 | 0.01-2.205 |  | 1.81 | 0.102-9.80 |  |
| **Subtype** |  | | |  | | |  | | |  | | |
| TNBC | 272 | 6.59-4318.99 | < 0.0005 | 3.96 | 0.675-15.00 | < 0.0005 | 0.5 | 0.03-1.71 | < 0.0005 | 1.85 | 0.102-5.232 | 0.47 |
| Non-TNBC | 306.85 | 5.74-14002.02 |  | 4.22 | 0.384-16.76 |  | 0.55 | 0.010-2.21 |  | 1.86 | 0.196-9.84 |  |
| **Receptor** |  | | |  | | |  | | |  | | |
| HR- | 287.05 | 6.46-4318.99 | < 0.0005 | 3.99 | 0.675-15.011 | < 0.0005 | 0.53 | 0.019-1.711 | < 0.0005 | 1.81 | 0.10-5.23 | 0.62 |
| HR+ | 305.33 | 5.74-14002.02 |  | 4.31 | 0.382-16.76 |  | 0.55 | 0.011-2.21 |  | 1.86 | 0.10-9.84 |  |
| **Stage** |  | | |  | | |  | | |  | | |
| Advanced | 336.14 | 6.46-14002.00 | 0.02 | 4.44 | 0.4216-16.763 | < 0.0005 | 0.55 | 0.0268-1.945 | 0.01 | 1.82 | 0.102-4.89 | 0.08 |
| Non- Advanced | 286.24 | 5.74-5201.00 |  | 4.11 | 0.384-16.769 |  | 0.53 | 0.106-2.205 |  | 1.88 | 0.201-9.84 |  |
