# Supplementary material for: Pan-immune-inflammation value: racial variations and differences in prognostic accuracy across breast cancer subtypes at a single institution
Source: Front Oncol. 2026 Mar 6;16:1694711. doi: 10.3389/fonc.2026.1694711 (PMC13002395; doi:10.3389/fonc.2026.1694711)
Supplement: Supplementary file 3 [file Table3.docx]

**Supplementary Table 3.** Cox regression modeling of overall survival.

| **Variable** | **Model 1** | | | **Model 2** | | | **Model 3** | | |
| --- | --- | --- | --- | --- | --- | --- | --- | --- | --- |
|  | **Exp(B)** | **95% CI** | **p-value** | **Exp(B)** | **95% CI** | **p-value** | **Exp(B)** | **95% CI** | **p-value** |
| **AJCC Stage** | 0.981 | 0.813-1.184 | **0.000** | 1.019 | 0.843-1.232 | **0.000** | 1.032 | 0.853-1.248 | **0.000** |
| **Age** | 1.017 | 1.010-1.024 | **0.042** | 1.016 | 1.009-1.024 | **0.043** | 1.016 | 1.009-1.023 | **0.048** |
| **Race** | 0.858 | 0.690-1.068 | 0.170 | 0.831 | 0.667-1.034 | 0.097 | 0.824 | 0.658-1.032 | 0.092 |
| **Subtype** | 0.918 | 0.573-1.471 | 0.722 | 0.908 | 0.566-1.454 | 0.687 | 0.799 | 0.483-1.332 | 0.383 |
| **HR Status** | 1.071 | 0.696-1.648 | 0.755 | 1.059 | 0.688-1.630 | 0.795 | 1.188 | 0.746-1.891 | 0.469 |
| **PIV** | Not included | - | - | 1.000 | 1.000-1.000 | **0.000** | 1.000 | 1.000-1.001 | 0.324 |
| **PIV*Race** | Not included | - | - | Not included | - | - | 1.000 | 0.999-1.001 | 0.797 |
| **PIV*Subtype** | Not included | - | - | Not included | - | - | 0.999 | 0.999-1.000 | **0.024** |
| **PIV*HR Status** | Not included | - | - | Not included | - | - | 1.001 | 1.000-1.002 | **0.000** |
