# Supplementary figures and images for: Pan-immune-inflammation value: racial variations and differences in prognostic accuracy across breast cancer subtypes at a single institution
Source: Front Oncol. 2026 Mar 6;16:1694711. doi: 10.3389/fonc.2026.1694711 (PMC13002395; doi:10.3389/fonc.2026.1694711)

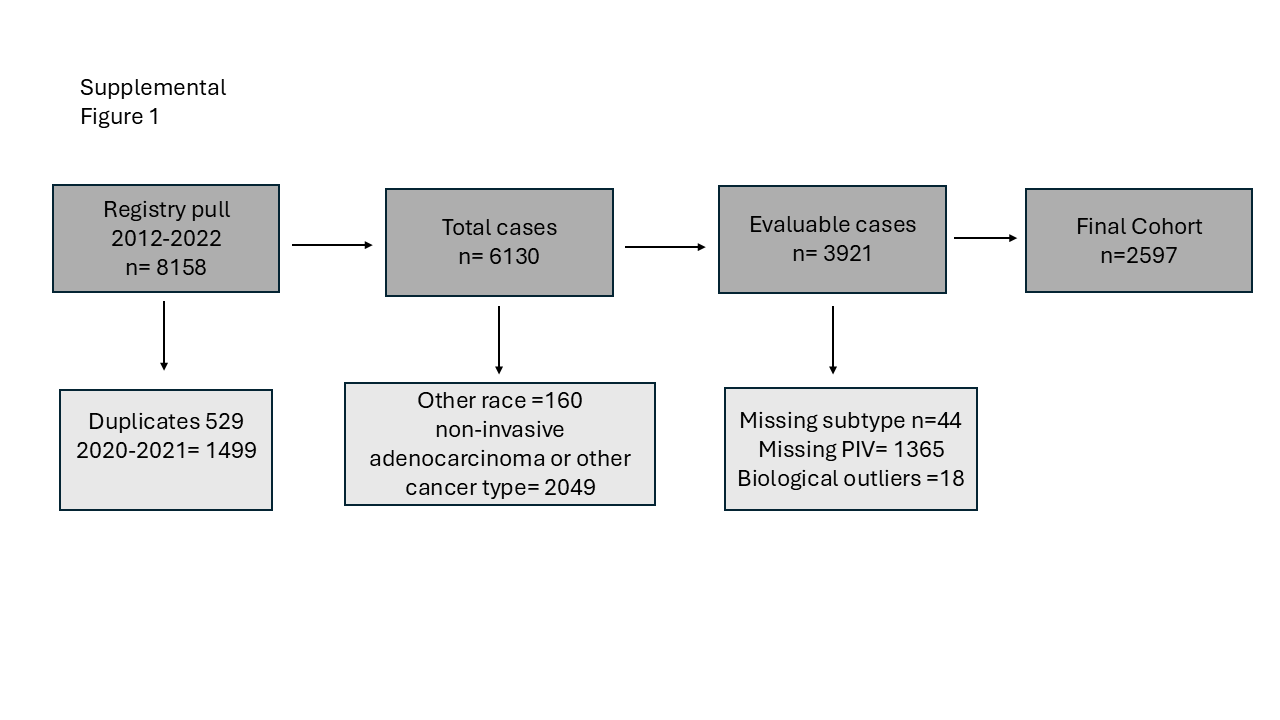

Supplement: Supplementary Table 1 — Patient clinical characteristics. [file Image1.tif]

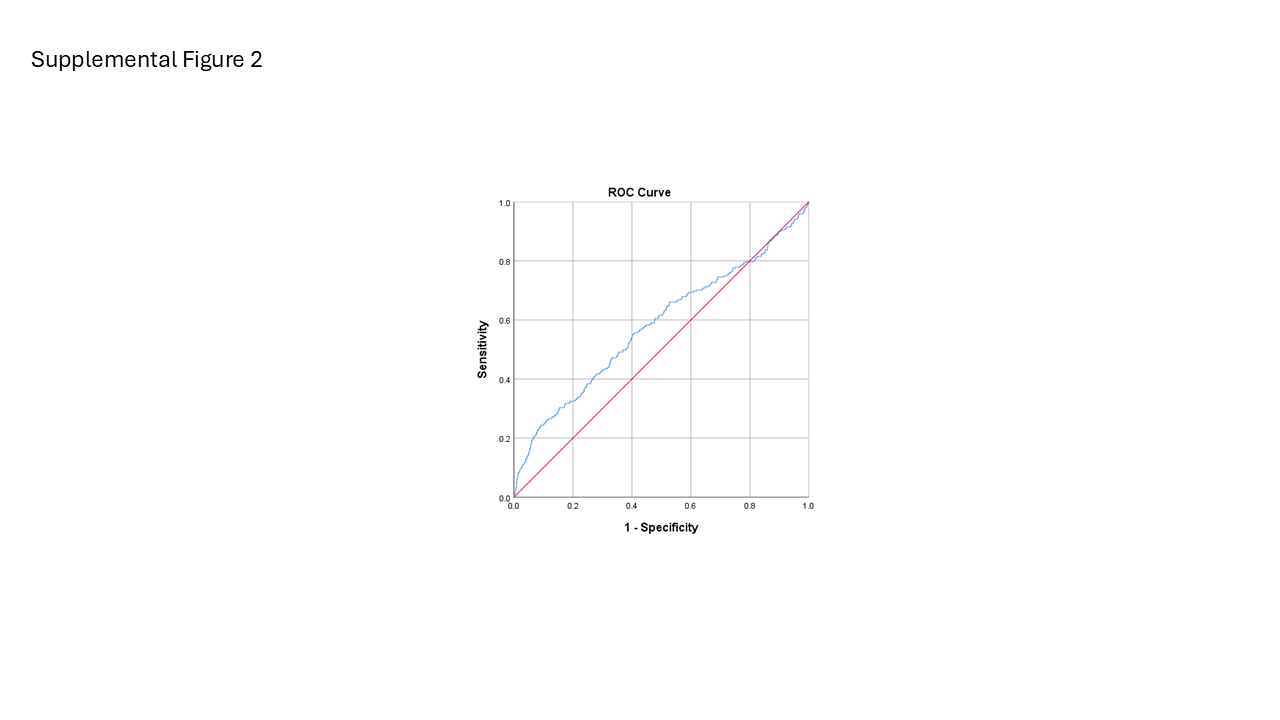

Supplement: Supplementary Table 2 — Pan-Immune-Inflammatory-Value (PIV) and immune cell levels by patient characteristics. Immune cell values given at 103 cells/µL. [file Image2.tif]

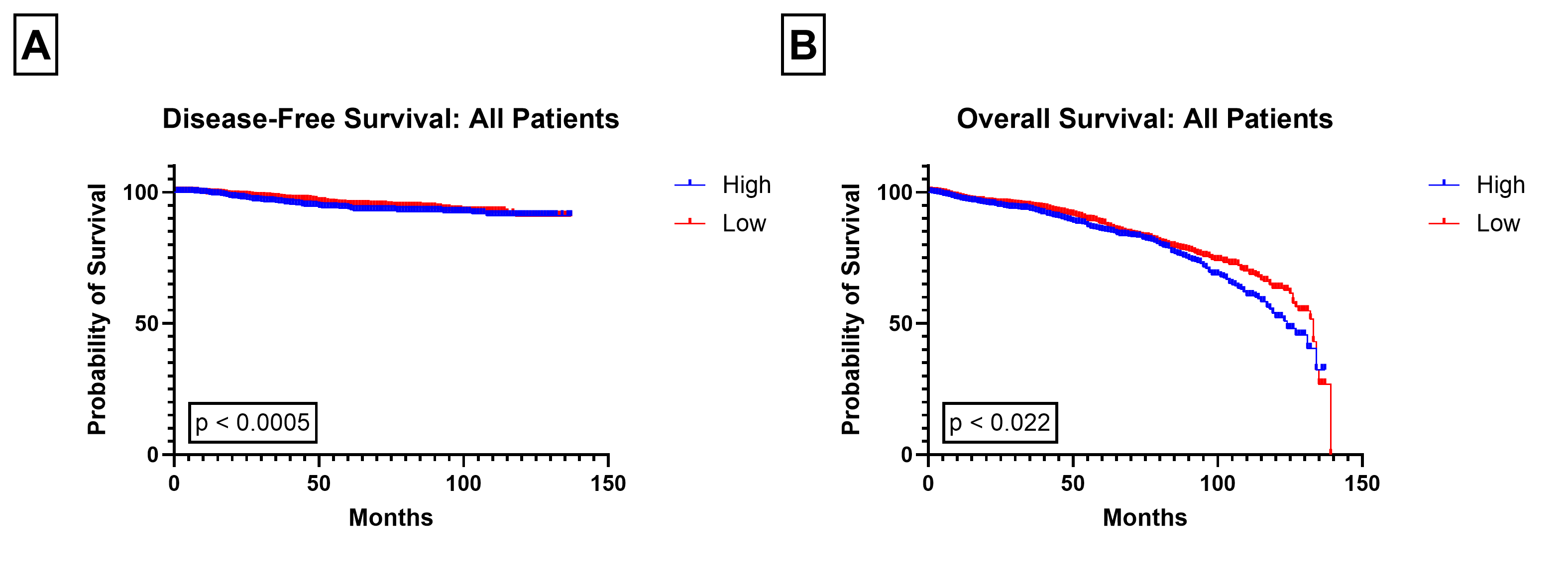

Supplement: Supplementary Table 3 — Cox regression modeling of overall survival. [file Image3.tif]

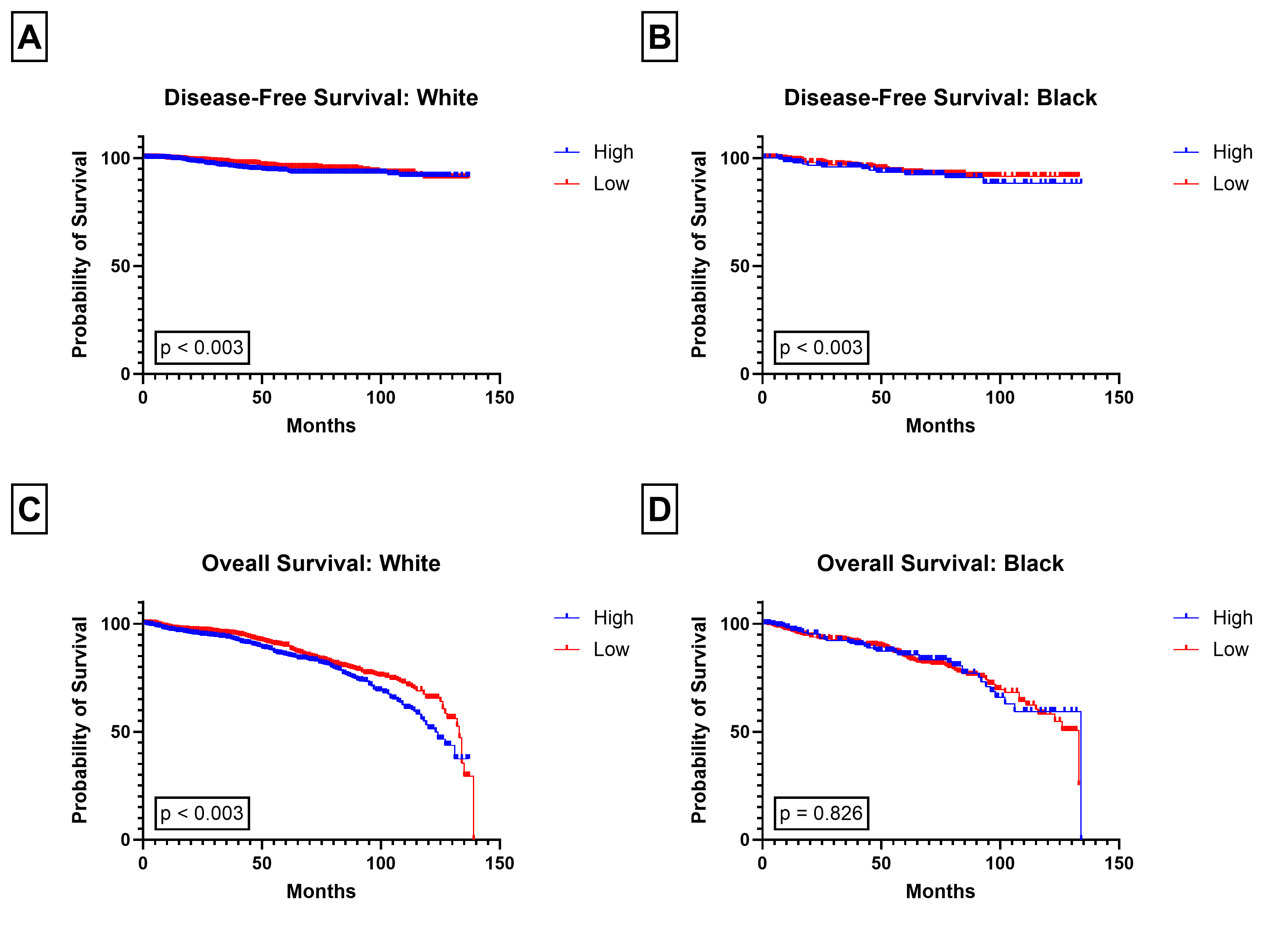

Supplement: Supplementary Figure 1 — Patient exclusion flowchart for cohort selection. [file Image4.tif]

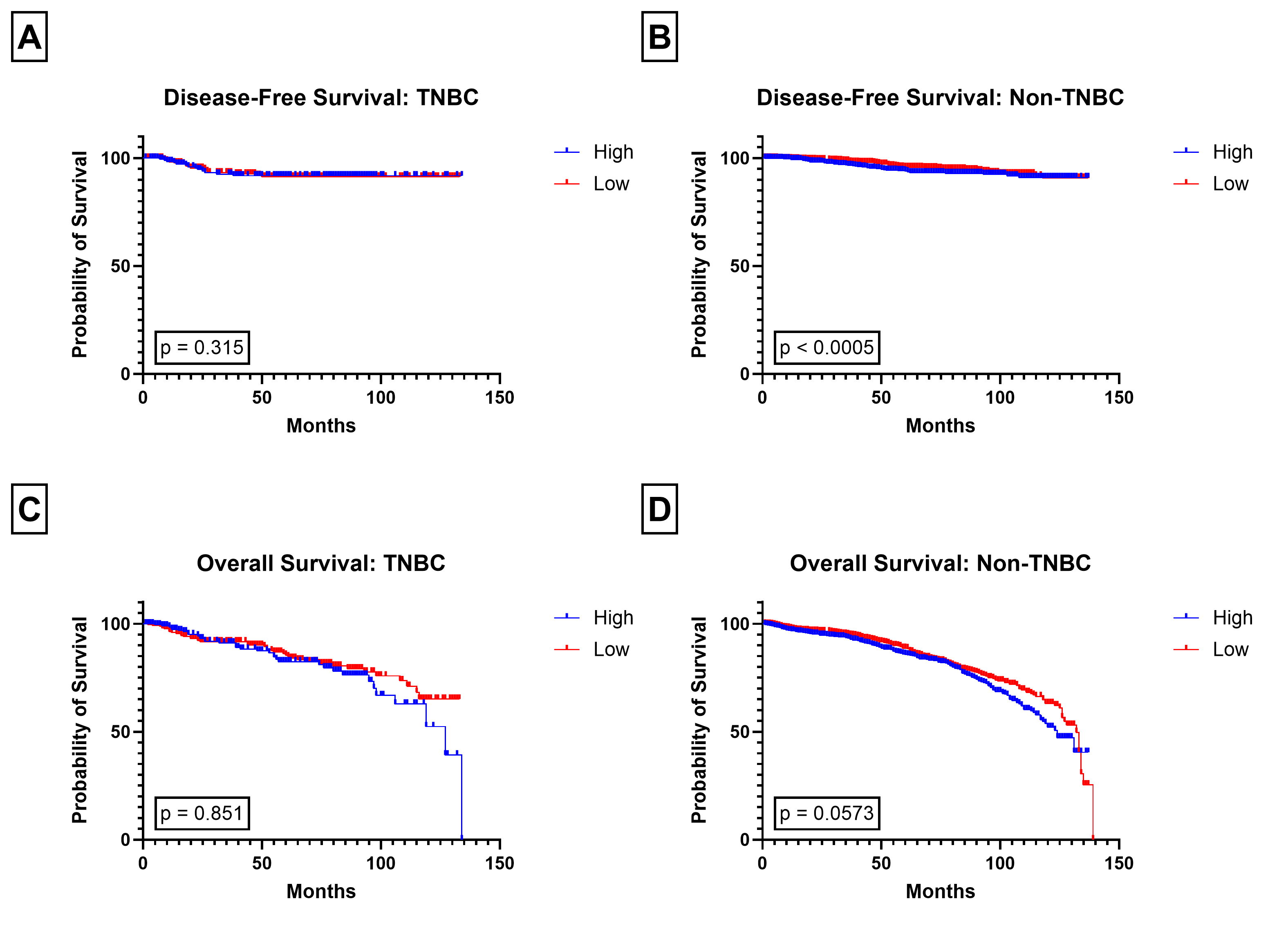

Supplement: Supplementary Figure 2 — Receiver operating characteristic curve (ROC) for optimal PIV cut-off threshold. [file Image5.tif]
